# Meteorological Report for April

**Published:** 1879-05

**Authors:** 


### METEOROLOGICAL REPORT FOR APRIL.

| RAIN. DATE | RAIN. DAY. | Mean daily Barometer. | Mean daily Thermometer | Mean daily Humidity. | Maximum Thermometer | Minimum Thermometer | Prevailing direction. |
|---|---|---|---|---|---|---|---|
| 1 | 1.14 | 29.742 | 49.0 | 76.7 | 56 | 45 | N. W. |
| 2 | 0 | 29.907 | 49.2 | 50.0 | 56 | 43 | N. W. |
| 3 | 0 | 29.994 | 43.7 | 46.0 | 53 | 37 | N. W. |
| 4 | 0 | 30.069 | 40.2 | 38.3 | 48 | 30 | N. W. |
| 5 | 0 | 30.124 | 45.7 | 38.3 | 53 | 34 | N. W. |
| 6 | 0 | 30.113 | 53.2 | 33.0 | 64 | 38 | S. |
| 7 | .19 | 29.930 | 56 2 | 72.0 | 62 | 50 | S. |
| 8 | 0 | 30.092 | 55.0 | 57.3 | 63 | 47 | N. E. |
| 9 | 0 | 29.944 | 58.0 | 52.5 | 65 | 46 | E. |
| 10 | .18 | 29.628 | 63.2 | 55 3 | 70 | 59 | S. W. |
| 11 | 0 | 29.864 | 55.2 | 55.3 | 62 | 52 | W. |
| 12 | 0 | 30.091 | 58.0 | 47.3 | 66 | 42 | N. |
| 13 | 0 | 29 956 | 66.7 | 53.7 | 75 | 50 | S. |
| 14 | .15 | 29.735 | 65.0 | 74.7 | 71 | 57 | S. & E. |
| 15 | .02 | 29 727 | 66.5 | 74.6 | 73 | 61 | S. W. |
| 16 | 1.59 | 29.597 | 64.0 | 91.0 | 69 | 58 | S. E. |
| 17 | .22 | 29 692 | 53.0 | 73.0 | 65 | 50 | N. W. |
| 18 | 0 | 29.984 | 51.5 | 46.7 | 58 | 43 | N. W. |
| 19 | 0 | 29 985 | 60.0 | 38.0 | 67 | 45 | W. |
| 20 | 0 | 30.033 | 59 0 | 45.3 | 69 | 45 | N. |
| 21 | 0 | 30.195 | 65 0 | 41.3 | 74 | 50 | E. |
| 22 | 0 | 30.373 | 66.7 | 43.3 | 75 | 55 | E. |
| 23 | 0 | 30 285 | 69.5 | 40.0 | 76 | 57 | S. E. |
| 24 | 0 | 30.145 | 71.2 | 39 0 | 80 | 59 | S. |
| 25 | 0 | 30.063 | 71.0 | 38.3 | 79 | 57 | S. |
| 26 | 0 | 29.967 | 70.0 | 50.0 | 78 | 60 | S. E. |
| 27 | .48 | 29.960 | 62.7 | 83.0 | 69 | 61 | S. E. |
| 28 | .01 | 29.930 | 66.7 | 71.7 | 75 | 61 | N. W. |
| 29 | 0 | 29.936 | 68.7 | 43.7 | 78 | 58 | N. W. |
| 30 | 0 | 29.895 | 70.7 | 38.3 | 79 | 57 | N. W. |
| Monthly Means. | | 29.895 | 59.8 | 53.6 | 67.6 | 50.2 | N. W. |

Highest Barometer, 30.409 on the 22d, at 7 and 7.06 A.M.
Lowest Barometer, 29.548, on the 16th, at 2 P.M.
Monthly range of Barometer, 0.861.
Highest Temperature, 80°, on 24th. Lowest Temperature, 30°, on 4th.
Monthly range of Temperature, 50°.
Greatest daily range of Temperature, 26°, on the 1st 6th.
Least daily range of Temperature, 8°, on the 27th.
Mean of Maximum Temperatures, 67°6.
Mean of Minimum Temperatures, 50°2.
Mean daily range of Temperature, 17°4.
Total rainfall, 3.98 inches.
Prevailing wind, Northwest. Total movement of wind, 78.06 miles.
Maximum velocity of wind and direction, 34 miles per hour, Northwest and East, on the 3d and 16th.
*Number of Foggy days, none. Number of Cloudy days on which rain fell, 7.
Number of cloudy days on which no rain fell, 3.
Total number of days on which rain fell, 9.

